# Association between sleep, sunlight exposure, and multimorbidity in older adults with and without mental illness

**DOI:** 10.3389/fpubh.2026.1751563

**Published:** 2026-03-10

**Authors:** Nan Zhang, Jingpeng Gao, Yajie Che, Cui Wang, Shanshan Chen, Shan Yu, Miao Miao, Ping Yan, Siyuan Tang

**Affiliations:** 1 Xiangya School of Nursing, Central South University, Changsha, China; 2 School of Nursing, Xinjiang Medical University, Urumqi, China; 3 Health Care Research Center for Xinjiang Regional Population, Urumqi, China; 4 Department of Infectious Disease, General Hospital of Xinjiang Military Command, Urumqi, China; 5 School of Nursing, Zhejiang Chinese Medical University, Hangzhou, China; 6 Xinjiang Key Laboratory of Mental Development and Learning Science, School of Psychology, Xinjiang Normal University, Urumqi, China

**Keywords:** mental disorder, multimorbidity, older people, sleep, sunlight

## Abstract

**Background:**

Light exposure and sleep are closely related to many chronic conditions. However, the associations between light exposure, sleep, and multimorbidity have been less well characterized, particularly in older adults with and without mental illness.

**Methods:**

A cross-sectional study was conducted in two representative areas in Northwest China. Core sleep parameters, sleep-related symptoms, and sunlight exposure were collected using self-reported questionnaires. We clustered overall multimorbidity into three categories: physical-only, mental-only, and mental-physical multimorbidity. Separate multivariable binary logistic regression analyses were performed to examine the associations between sleep, sunlight exposure, and multimorbidity.

**Results:**

Of the 1,018 participants, the mean age was 68.56 years, and 48.53% were female. Approximately half of the participants had two or more chronic conditions, including physical-only (21.22%), mental-only (1.57%), and mental-physical (28.49%) multimorbidity. Adjusted odds ratios (AORs) for sunlight exposure were significantly lower in older adults with mental-physical multimorbidity compared with those without. Higher odds for poor sleep quality, insomnia, snoring, and daytime sleepiness were observed in older adults with either category of multimorbidity. Sleep duration, non-extreme sleep timing, and sleep efficiency were associated with reduced odds for overall and mental-physical multimorbidity. Stratified analyses demonstrated stronger associations between sleep with multimorbidity in females and the younger-old group, whereas sunlight exposure was inversely associated with multimorbidity only in males.

**Conclusion:**

Multimorbidity was prevalent in community-dwelling older adults. Adequate sunlight exposure and good sleep quality are associated with reduced odds of mental-physical multimorbidity.

## 1 Introduction

Multimorbidity, defined as the co-occurrence of two or more long-term conditions in an individual, represents a substantial and growing public health challenge (1). In contrast to comorbidity, which is a disease-focused concept that emphasizes additional conditions in relation to an index condition, multimorbidity represents a broader, more holistic, and patient-centered framework that conceptualizes the total number and types of conditions a person has (1, 2). The prevalence of multimorbidity increases sharply with age, affecting up to 62% of individuals aged 65–74 years and 81.5% of those aged 85 years and older (2). Multimorbidity is linked to elevated risks of mortality, disability, functional decline, and diminished quality of life (1, 3, 4). It also imposes a heavy economic burden on healthcare systems globally through increased healthcare service utilization and catastrophic health expenditures (5, 6).

Accumulating evidence suggests that mental and physical health problems not only share common genetic correlations but also act synergistically to exert negative effects on disability, leading to prolonged hospitalizations, higher costs, and increased mortality (4, 7, 8). Several studies have established associations between mental health problems (e.g., depression, anxiety) and various physical conditions, including dementia, cardiovascular diseases, musculoskeletal disorders, and gastroesophageal diseases (9, 10). A recent meta-analysis reported that people with mental health conditions have 1.84-fold higher odds (95% CI: 1.33–2.54) of having physical multimorbidity compared to those without (11). Furthermore, individuals with mental-physical multimorbidity exhibit more than a 150% increase in the number of outpatient visits and hospitalization days, and face a 2.2-fold higher likelihood of catastrophic health expenditure (5). Among those with common mental illness, each additional physical illness elevates the risk of frequent visits to the emergency department; indeed, the adjusted odds ratio increases from 2.08 (for mental illness alone) to 9.86 (for more than four physical conditions alongside mental illness), compared to individuals with neither condition (12). Thus, individuals with co-occurring mental and physical multimorbidity appear to constitute a distinct and high-risk phenotype.

Non-optimal sleep patterns, encompassing impaired sleep parameters (e.g., abnormal sleep duration, evening chronotype, irregular sleep, low sleep efficiency) and sleep-related symptoms (e.g., sleep disturbance, insomnia, daytime sleepiness, snoring) are crucial modifiable risk factors that are positively associated with multimorbidity (13–16). However, prior research has largely focused on the relationship between sleep and physical conditions, frequently overlooking the role of mental health within the multimorbidity spectrum. This limitation implies that adequate sleep alone may be insufficient to mitigate certain health risks, especially in the context of mental-physical multimorbidity, thereby highlighting the potential importance of additional modifiable environmental factors. Among these, sunlight exposure has emerged as a potentially significant contributor to overall health and well-being (17–19).

Light signals serve as fundamental zeitgebers (environmental cues that synchronize circadian rhythms), enabling photosensitive life forms to maintain stable yet adaptable circadian rhythms (20). Light–dark conditions can help peripheral clocks remain synchronized, even independently of the central clock mechanism in the suprachiasmatic nucleus (21, 22). When an individual’s lifestyle pattern becomes misaligned with the natural light–dark cycle, the risk of cardiovascular disease, metabolic syndrome, neurological conditions, and cancer may subsequently increase (21). Empirical evidence supports this viewpoint. A study of more than 85,000 individuals showed that day and night light exposure are associated with several mental illnesses (18). Another large study of over 400,000 UK Biobank participants demonstrated that greater time spent in daytime outdoor light was associated with improved mood, sleep, and circadian-related outcomes (19). Moreover, a recent large-scale study by Ma et al. (17) involving 362,094 participants, revealed a J-shaped relationship between sunlight exposure and dementia risk, wherein both insufficient and excessive sunlight exposure were associated with an increased risk, and an average of 1.5 h per day was associated with the lowest risk. Collectively, these evidence suggest that habitual light exposure may represent an environmental risk factor influencing not only mental health but also broader adverse health outcomes, potentially extending to multimorbidity.

Despite the established co-occurrence of physical and mental conditions, the associations between sunlight exposure, sleep, and multimorbidity in older adults remain poorly understood (3, 9). To date, no study has comprehensively investigated these relationships. Substantially less is known about the role of these factors in older adults with pre-existing mental illness. It is also unknown whether these relationships exert consistent effects in individuals with and without mental illness.

The primary aims of this study were: (1) to assess the prevalence of multimorbidity (overall, physical-only, mental-only, and mental-physical) among community-dwelling older adults in Northwest China; and (2) to examine the associations of sleep (including both sleep-related symptoms and parameters) and sunlight exposure with multimorbidity, and to investigate whether these associations differed between individuals with and without mental illness.

## 2 Methods

### 2.1 Study design and population

We conducted a cross-sectional study to investigate the prevalence of multimorbidity and its associations with sleep and sunlight exposure. Participants were recruited from two geographic regions in Xinjiang, China (Yarkant County, YK; Yili County, YL). The sites were selected to capture socioeconomic and environmental diversity among older adults in Northwest China. Participants were identified through a multistage, community-based sampling approach in collaboration with local community health centers and village committees, which provided registries of age-eligible residents and invited them to participate. Inclusion criteria were community-dwelling adults aged ≥60 years who were able to communicate in Mandarin or Uyghur. Exclusion criteria included (1) severe visual or hearing impairments and (2) severe medical conditions that precluded participation. This study was approved by the Research Ethics Committee of Xinjiang Medical University (IRB code: XJYKDXR20220725029). Face-to-face interviews were subsequently conducted at community health stations. Prior to enrollment, all participants received a detailed verbal and written explanation of the study’s purpose, procedures, risks, benefits, and their rights from a trained bilingual investigator. The informed consent process was conducted in the participant’s preferred language (Mandarin or Uyghur). All participants then provided written informed consent by signature or thumbprint (if illiterate) in the presence of a witness from local healthcare settings. Data were collected by trained investigators. Participants would receive 10 yuan as compensation for their time.

### 2.2 Sleep parameters and sleep-related symptoms

Sleep health is a multidimensional concept. Accordingly, we selected key sleep parameters based on the RU-SATED framework (23), choosing sleep duration, timing, regularity, and efficiency to represent the essential domains of sleep health. Participants were asked about their bedtime and wake-up time on workdays and weekends, sleep onset time, and whether they had a regular sleep pattern. The following parameters were derived: sleep duration (average time from sleep onset to final awakening), sleep timing (mean midpoint between sleep onset and awakening, where a midpoint outside of 2:00–4:00 a.m. was defined as an extreme sleep midpoint), sleep irregularity (self-reported irregular sleep–wake schedules), and sleep efficiency (self-reported total sleep time divided by time in bed, multiplied by 100%).

We also assessed the following sleep-related symptoms: sleep disturbance, insomnia, snoring, and daytime sleepiness. Sleep disturbance was measured using the Pittsburgh Sleep Quality Index (PSQI), with a score > 7 indicating poor sleep quality (24). Insomnia was assessed using the Insomnia Severity Index (ISI), using a cutoff score of > 10 (25). Snoring was evaluated by asking participants about the frequency of snoring or apnea (defined as cessation of breathing for ≥10 s) during sleep, which was categorized as never or rarely (≤ 1 time per week), occasionally (2–3 times per week), or frequently (≥ 4 times per week). Daytime sleepiness was determined by the Epworth Sleepiness Scale, where a score ≥ 11 indicated excessive sleepiness (26).

### 2.3 Sunlight exposure

Sunlight exposure time was collected using two self-reported questions: On average, (1) how much time do you spend outdoors under daylight (i.e., without overhead covering) on a typical workday? and (2) on a typical non-workday (rest day/weekend)? The average daily sunlight exposure was then calculated using the following formula: (workday exposure × 5 + non-workday exposure × 2)/7.

### 2.4 Definition of multimorbidity

Multimorbidity was defined as the coexistence of two or more chronic diseases in the same individual. A total of 14 chronic conditions were assessed, including hypertension; diabetes; heart disease or cardiovascular disease (CVD); stroke; cancer; chronic lung diseases (bronchitis, emphysema, pneumonia, asthma); liver diseases and hepatitis; kidney diseases (e.g., chronic nephritis); stomach or other digestive diseases (gastric or duodenal ulcer, cholecystitis, cholelith disease); emotional, nervous, or psychiatric problems; memory-related diseases (dementia, Parkinson’s disease, epilepsy); arthritis, rheumatism or rheumatoid disease; tuberculosis or AIDS; and peripheral vascular disease. The number of chronic diseases was summed for each participant, and those with two or more chronic diseases were classified as having multimorbidity.

Among these, we further categorized multimorbidity into three mutually exclusive subtypes: (1) physical-only multimorbidity, defined as the presence of ≥2 physical chronic conditions and no mental illness, as defined below; (2) mental-only multimorbidity, defined as the presence of ≥2 mental health conditions and no physical chronic conditions; and (3) mental-physical multimorbidity, defined as the coexistence of at least one physical chronic condition and at least one mental illness. Mental illness was defined as meeting any of the following criteria: (1) self-reported emotional, nervous, or psychiatric problems; (2) depression (indicated by a Geriatric Depression Scale-15 score ≥5) (27); or (3) anxiety (indicated by a Generalized Anxiety Disorder-7 score≥5) (28).

These multimorbidity categories were analyzed as separate, mutually exclusive, binary outcomes to explore differential associations with sleep characteristics and sunlight exposure. A separate mental-only multimorbidity category (i.e., two or more mental health conditions without any physical chronic condition) was not included in the analysis because only 16 participants (1.57% of the sample) met this criterion, which was insufficient to support reliable multivariable regression modeling.

### 2.5 Covariates

The following covariates were controlled for the analysis: age (years), gender (male/female), cognitive function (Montreal Cognitive Assessment, MoCA, score), education (illiterate/primary school/high school), marital status (married/others including widowed, divorced, or single), monthly per-capita household income (<1000/1000–1999/≥2000), smoking status (current smokers/others), drinking status (current drinkers/others), and body mass index (BMI).

### 2.6 Statistical analysis

Variables were described using means and standard deviations (SDs) for continuous variables, and frequencies with percentages for categorical variables. For continuous variables, comparisons were performed using the t-test for two independent groups. For categorical variables, Chi-square tests or Fisher’s exact tests were utilized to analyze the proportions between different groups. For inferential analyses, separate multivariable binary logistic regression models were fitted for overall, physical-only, and mental-physical multimorbidity. Mental-only multimorbidity was not modeled due to the small sample size (*n* = 16). This approach was selected because the primary aim was to examine associations with specific multimorbidity patterns, and the small number of mental-only cases precluded stable multinomial modeling. Bootstrap resampling with 1,000 replications was applied to obtain robust odds ratios and 95% confidence intervals, thereby reducing the influence of potential non-normality and model instability. We applied four hierarchical models to explore the association between sleep/sunlight exposures and multimorbidity outcomes, with sequential adjustment for covariates across different conceptual levels, as previously described (29). The first model was a crude model without adjustment for any covariate; Model 1 adjusted for individual factors (gender, age, cognitive function); Model 2 additionally adjusted for societal-level covariates (education level, marital status, household income); and Model 3 further adjusted for behaviors/physiology-level covariates (smoking status, drinking status, BMI). The results are reported as bootstrap-estimated adjusted odds ratios (AORs) with 95% confidence intervals (CIs). All statistical analyses were performed in Stata 15.1, with statistical significance set at a two-tailed alpha of 0.05 or less.

## 3 Results

### 3.1 General characteristics of participants

Of the 1,018 participants, 524 (51.47%) were male and 494 (48.53%) were female. The mean age was 69.30 years (SD = 6.60) for males and 67.77 years (SD = 6.30) for females. Compared with male participants, female participants had a higher prevalence of illiteracy, were more likely to be widowed, divorced, or single, and had poorer cognitive performance, as well as lower rates of smoking and drinking. Additionally, female participants reported significantly less sunlight exposure, greater sleep disturbance, more pronounced insomnia symptoms, a higher prevalence of sleep irregularity, and lower sleep efficiency. Detailed participant characteristics are summarized in Table 1.

**Table 1:** General characteristics of the study population.

Variables	Total	Male	Female	*p*
N	1,018	524	494	
Age (year)	68.56 ± 6.50	69.30 ± 6.60	67.77 ± 6.30	<0.001
Education level, n (%)				0.005
Illiteracy	318 (31.24)	140 (26.72)	178 (36.03)	
Primary school	513 (50.39)	285 (54.39)	228 (46.15)	
High school	187 (18.37)	99 (18.89)	88 (17.82)	
Income, n (%)				0.444
<1000 yuan	822 (80.75)	416 (79.39)	406 (82.19)	
1,000–1999 yuan	107 (10.51)	61 (11.64)	46 (9.31)	
≥2000 yuan	89 (8.74)	47 (8.97)	42 (8.50)	
Marital status, n (%)				<0.001
Married	774 (76.03)	446 (85.11)	328 (66.40)	
Others	244 (23.97)	78 (14.89)	166 (33.60)	
Cognitive function	15.11 ± 5.64	15.89 ± 5.40	14.27 ± 5.77	<0.001
Body mass index	26.19 ± 4.71	26.23 ± 4.60	26.14 ± 4.83	0.748
Smoking, n (%)				<0.001
Current smokers	118 (11.59)	111 (21.18)	7 (1.42)	
Others	900 (88.41)	413 (78.82)	487 (98.58)	
Drinking, n (%)				<0.001
Drinkers	56 (5.50)	53 (10.11)	3 (0.61)	
Non-drinkers	962 (94.50)	471 (89.89)	491 (99.39)	
Sunlight exposure (h)	3.59 ± 2.00	3.90 ± 2.14	3.27 ± 1.78	<0.001
Sleep-related symptoms				
PSQI score	6.91 ± 3.68	6.32 ± 3.41	7.54 ± 3.85	<0.001
Sleep disturbance (PSQI> 7)	391 (38.41)	160 (30.53)	231 (46.76)	<0.001
ISI score	8.71 ± 7.23	7.76 ± 6.72	9.71 ± 7.61	<0.001
Clinical insomnia (ISI > 10)	330 (32.42)	138 (26.34)	192 (38.87)	<0.001
Snoring				0.223
Never or rarely snoring	644 (63.26)	336 (64.12)	308 (62.35)	
Sometimes snoring	234 (22.99)	110 (20.99)	124 (24.29)	
Often snoring	140 (13.75)	78 (14.89)	62 (12.55)	
ESS score	5.71 ± 4.23	5.75 ± 4.21	5.66 ± 4.21	0.727
Daytime sleepiness (ESS ≥ 11)	125 (12.28)	63 (12.02)	62 (12.55)	0.798
Sleep parameters				
Sleep duration	7.29 ± 1.52	7.29 ± 1.54	7.29 ± 1.50	0.976
Sleep timing	231 (22.69)	117 (22.33)	114 (23.08)	0.776
Sleep irregularity	263 (25.83)	121 (23.09)	141 (28.54)	0.039
Sleep efficiency	0.90 ± 0.12	0.90 ± 0.12	0.88 ± 0.13	0.025

Marital status “Others” (i.e., widowed, divorced, or single).

### 3.2 Prevalence of overall multimorbidity, physical-only multimorbidity, and mental-physical multimorbidity

The prevalences of overall multimorbidity, physical-only multimorbidity, mental-only multimorbidity, and mental-physical multimorbidity are presented in Table 2. In our study, half of older adults (522 participants, 51.28%) had two or more chronic conditions. The prevalences of physical-only multimorbidity, mental-only multimorbidity, and mental-physical multimorbidity were 21.22, 1.57, and 28.49%, respectively.

**Table 2:** Prevalence of sleep, sunlight exposure, and multimorbidity among the study population.

Variables	Overall multimorbidity (≥2 chronic conditions)			Physical-only multimorbidity (≥2 physical conditions, without mental illness)			Mental-only multimorbidity (≥2 mental health conditions, without physical conditions)			Mental-physical multimorbidity (co-occurrence of physical and mental conditions)		
	Yes	No	*p*	Yes	No	*p*	Yes	No	*p*	Yes	No	*p*
Total, n (%)	522 (51.28)	496 (48.72)		216 (21.22)	802 (78.78)		16 (1.57)	1,002 (98.43)		290 (28.49)	728 (71.51)	
Gender, n (%)			<0.001			0.558			0.131			<0.001
Male	225 (43.10)	299 (60.28)		115 (53.24)	409 (51.00)		5 (31.25)	519 (51.80)		105 (36.21)	419 (57.55)	
Female	297 (56.90)	197 (39.72)		101 (46.76)	393 (49.00)		11 (68.75)	483 (48.20)		185 (63.79)	309 (42.45)	
Age (year)	68.80 ± 6.69	68.30 ± 6.29	0.222	69.85 ± 7.09	68.21 ± 6.29	0.001	65.19 ± 6.20	68.61 ± 6.49	0.037	68.22 ± 6.27	68.69 ± 6.59	0.294
60–69	297 (56.90)	306 (61.69)	0.120	106 (49.07)	497 (61.97)	0.001	13 (81.25)	590 (58.88)	0.078	178 (61.38)	425 (58.38)	0.379
≥70	225 (43.10)	190 (38.31)		110 (50.93)	305 (38.03)		3 (18.75)	412 (41.12)		112 (38.62)	303 (41.62)	
Education level, n (%)			0.480			0.991			0.999			0.226
Illiteracy	172 (32.95)	146 (29.44)		65 (30.09)	253 (31.55)		5 (31.25)	313 (31.24)		102 (35.17)	216 (29.67)	
Primary school	257 (49.23)	256 (51.61)		110 (50.93)	403 (50.25)		8 (50.00)	505 (50.40)		139 (47.93)	374 (51.37)	
High school	93 (17.82)	94 (18.95)		41 (18.98)	146 (18.20)		3 (18.75)	184 (18.36)		49 (16.90)	138 (18.96)	
Income, n (%)			0.272			0.152			0.441			0.296
<1000 yuan	428 (82.00)	394 (79.43)		173 (80.10)	649 (80.92)		12 (75.00)	810 (80.84)		243 (83.79)	579 (79.53)	
1,000–1999 yuan	47 (9.00)	60 (12.10)		18 (8.33)	89 (11.10)		3 (18.75)	103 (10.25)		26 (8.97)	81 (11.13)	
≥2000 yuan	47 (9.00)	42 (8.47)		25 (11.97)	64 (7.98)		1 (6.25)	89 (8.88)		21 (7.24)	68 (9.34)	
Marital status, n (%)			0.568			0.224			0.384			0.042
Married	393 (75.29)	381 (76.81)		171 (79.17)	603 (75.19)		14 (87.50)	760 (75.85)		208 (71.72)	566 (77.75)	
Others	129 (24.71)	115 (23.19)		45 (20.83)	199 (24.81)		2 (12.50)	242 (24.15)		82 (28.28)	162 (22.25)	
Cognitive function	14.32 ± 5.76	15.94 ± 5.39	<0.001	15.40 ± 5.46	15.03 ± 5.68	0.394	15.79 ± 7.08	15.10 ± 5.61	0.624	13.44 ± 5.76	15.77 ± 5.45	<0.001
Body mass index	26.83 ± 4.96	25.51 ± 4.34	<0.001	27.70 ± 5.12	25.78 ± 4.52	<0.001	24.97 ± 3.43	26.20 ± 4.73	0.298	26.29 ± 4.81	26.14 ± 4.67	0.653
Smoking status, n (%)			0.492			0.343			0.241			0.223
Current smokers	57 (10.92)	61 (12.30)		29 (13.43)	89 (11.10)		0	118 (11.78)		28 (9.66)	90 (12.36)	
Others	465 (89.08)	435 (87.70)		187 (86.57)	713 (88.90)		16 (100.00)	884 (88.22)		262 (90.34)	638 (87.64)	
Drinking status, n (%)			0.637			0.968			1.000			0.772
Drinkers	27 (5.17)	29 (5.85)		12 (5.56)	44 (5.49)		0	56 (5.59)		15 (5.17)	41 (5.63)	
Non-drinkers	495 (94.83)	467 (94.15)		204 (94.44)	758 (94.51)		16 (100.00)	946 (94.41)		275 (94.83)	687 (94.37)	
Sunlight exposure (h)	3.10 ± 1.62	3.80 ± 2.05	<0.001	3.22 ± 1.59	3.50 ± 1.94	0.055	2.62 ± 1.45	3.45 ± 1.88	0.078	3.03 ± 1.64	3.60 ± 1.93	<0.001
Sleep-related symptoms												
PSQI score	8.65 ± 3.73	5.32 ± 3.21	<0.001	7.97 ± 3.51	6.77 ± 3.92	<0.001	9.19 ± 3.53	6.99 ± 3.86	0.024	9.12 ± 3.84	6.19 ± 3.55	<0.001
Sleep disturbance (PSQI>7)	292 (55.94)	99 (19.96)	<0.001	104 (48.15)	287 (35.79)	0.001	11 (68.75)	5 (31.25)	0.018	177 (61.03)	214 (29.40)	<0.001
ISI score	11.38 ± 7.02	4.80 ± 5.28	<0.001	9.73 ± 6.61	7.75 ± 7.10	<0.001	12.81 ± 5.15	8.10 ± 7.05	0.008	12.52 ± 7.18	6.44 ± 6.20	<0.001
Clinical insomnia (ISI > 10)	258 (49.43)	72 (14.52)	<0.001	83 (38.43)	247 (30.80)	0.034	11 (68.75)	319 (31.84)		164 (56.55)	166 (22.80)	<0.001
Snoring			<0.001			0.001			0.934			<0.001
Never or rarely snoring	251 (48.08)	393 (79.23)		100 (46.30)	544 (67.83)		11 (68.75)	633 (63.18)		140 (48.28)	504 (69.23)	
Sometimes snoring	161 (30.84)	73 (14.72)		66 (30.55)	168 (20.95)		3 (18.75)	231 (23.05)		92 (31.72)	142 (19.51)	
Often snoring	110 (21.08)	30 (6.05)		50 (23.15)	90 (11.22)		2 (12.50)	138 (13.77)		58 (20.00)	82 (11.26)	
ESS score	6.78 ± 4.28	4.58 ± 3.88	<0.001	6.56 ± 4.21	5.48 ± 4.21	0.001	8.50 ± 6.10	5.66 ± 4.19	0.008	6.85 ± 4.20	5.25 ± 4.16	<0.001
Daytime sleepiness (ESS ≥ 11)	90 (17.24)	35 (7.06)	<0.001	39 (18.06)	86 (10.72)	0.004	6 (37.5)	119 (11.88)	0.009	45 (15.52)	80 (10.99)	0.047
Sleep parameters												
Sleep duration	7.07 ± 1.51	7.48 ± 1.49	<0.001	7.19 ± 1.27	7.29 ± 1.57	0.432	6.56 ± 1.57	7.28 ± 1.51	0.060	7.00 ± 1.65	7.37 ± 1.44	<0.001
Sleeping timing	142 (27.20)	89 (17.94)	<0.001	54 (25.00)	177 (22.07)	0.361	7 (43.75)	224 (22.36)	0.017	81 (27.93)	150 (20.60)	0.012
Sleep irregularity	137 (26.25)	126 (25.40)	0.759	55 (25.46)	208 (25.94)	0.888	6 (37.5)	257 (25.65)	0.265	76 (26.21)	187 (25.69)	0.864
Sleep efficiency	0.87 ± 0.12	0.91 ± 0.11	<0.001	0.87 ± 0.12	0.89 ± 0.12	0.022	0.81 ± 0.16	0.89 ± 0.12	0.005	0.87 ± 0.13	0.90 ± 0.12	0.001

Values are presented as mean ± standard deviation (SD) or n (%). Multimorbidity subtypes were defined as mutually exclusive categories; therefore, the sum of physical-only, mental-only, and mental–physical multimorbidity equals the total number of individuals with overall multimorbidity.

### 3.3 Association between multimorbidity with sleep and sunlight exposure

The results of the multivariable logistic regression analysis are shown in Table 3. The associations between sunlight exposure and multimorbidity differed between older adults with and without mental illness. Specifically, each additional hour of daily sunlight exposure was associated with 17 and 14% lower odds of overall multimorbidity (AOR = 0.83, 95% CI: 0.77–0.90) and mental-physical multimorbidity (AOR = 0.86, 95% CI: 0.79–0.94), respectively, whereas this association was not significant for physical-only multimorbidity.

**Table 3:** Logistic regression model associations between sleep, sunlight exposure, and multimorbidity in older adults (N = 1,018).

Variables	Models	Overall multimorbidity (≥2 chronic conditions)		Physical-only multimorbidity (≥2 physical conditions)		Mental-physical multimorbidity (co-occurrence of physical and mental conditions)	
		AOR (95%CI)	*p*	AOR (95%CI)	*p*	AOR (95%CI)	*p*
Sunlight exposure time (hours)	Crude	0.80 (0.75, 0.86)	<0.001	0.92 (0.84, 0.99)	0.042	0.83 (0.76, 0.90)	<0.001
	Model 1	0.83 (0.77, 0.89)	<0.001	0.93 (0.85, 1.01)	0.071	0.86 (0.79, 0.94)	<0.001
	Model 2	0.83 (0.77, 0.89)	<0.001	0.92 (0.85, 1.00)	0.060	0.87 (0.80, 0.94)	0.001
	Model 3	0.83 (0.77, 0.90)	0.001	0.93 (0.85, 1.02)	0.107	0.86 (0.79, 0.94)	0.001
PSQI score	Crude	1.32 (1.26, 1.38)	<0.001	1.08 (1.04, 1.12)	<0.001	1.22 (1.18, 1.27)	<0.001
	Model 1	1.31 (1.25, 1.37)	<0.001	1.09 (1.05, 1.13)	<0.001	1.21 (1.16, 1.26)	<0.001
	Model 2	1.31 (1.25, 1.38)	<0.001	1.09 (1.05, 1.13)	<0.001	1.21 (1.16, 1.26)	<0.001
	Model 3	1.30 (1.24, 1.37)	<0.001	1.08 (1.04, 1.12)	0.026	1.21 (1.16, 1.26)	<0.001
ISI score	Crude	1.19 (1.16, 1.22)	<0.001	1.04 (1.02, 1.06)	<0.001	1.13 (1.11, 1.16)	<0.001
	Model 1	1.19 (1.16, 1.23)	<0.001	1.04 (1.02, 1.06)	<0.001	1.13 (1.10, 1.16)	<0.001
	Model 2	1.20 (1.17, 1.23)	<0.001	1.04 (1.02, 1.06)	<0.001	1.13 (1.10, 1.15)	<0.001
	Model 3	1.19 (1.16, 1.23)	<0.001	1.04 (1.02, 1.06)	<0.001	1.13 (1.11, 1.16)	<0.001
Sometimes snoring (referred to as never or rarely snoring)	Crude	3.45 (2.47, 4.82)	<0.001	2.14 (1.47, 3.11)	<0.001	2.33 (1.68, 3.24)	<0.001
	Model 1	3.53 (2.49, 5.00)	<0.001	2.25 (1.54, 3.29)	<0.001	2.27 (1.61, 2.82)	<0.001
	Model 2	3.60 (2.52, 5.12)	<0.001	2.23 (1.53, 3.27)	<0.001	2.34 (1.65, 3.31)	<0.001
	Model 3	3.52 (2.46, 5.02)	<0.001	2.13 (1.44, 3.13)	<0.001	2.38 (1.67, 3.37)	<0.001
Often snoring (referred to never or rarely snoring)	Crude	5.74 (3.61, 9.12)	<0.001	3.02 (1.98, 4.61)	<0.001	2.55 (1.75, 3.70)	<0.001
	Model 1	6.53 (4.04, 10.56)	<0.001	3.30 (2.15, 5.05)	<0.001	2.71 (1.81, 4.07)	<0.001
	Model 2	6.55 (4.03, 10.65)	<0.001	3.24 (2.10, 5.00)	<0.001	2.76 (1.82, 4.17)	<0.001
	Model 3	5.96 (3.64, 9.76)	<0.001	2.77 (1.75, 4.39)	<0.001	2.83 (1.86, 4.31)	<0.001
ESS score	Crude	1.14 (1.11, 1.18)	<0.001	1.06 (1.03, 1.10)	<0.001	1.10 (1.06, 1.13)	<0.001
	Model 1	1.14 (1.12, 1.18)	<0.001	1.07 (1.03, 1.11)	<0.001	1.08 (1.05, 1.12)	<0.001
	Model 2	1.14 (1.11, 1.18)	<0.001	1.07 (1.03, 1.11)	<0.001	1.08 (1.06, 1.12)	<0.001
	Model 3	1.13 (1.10, 1.17)	<0.001	1.06 (1.02, 1.09)	0.003	1.09 (1.05, 1.13)	<0.001
Sleep duration	Crude	0.83 (0.76, 0.90)	<0.001	0.96 (0.88, 1.05)	0.371	0.85 (0.77, 0.93)	<0.001
	Model 1	0.83 (0.76, 0.90)	<0.001	0.95 (0.87, 1.04)	0.261	0.85 (0.77, 0.94)	0.002
	Model 2	0.83 (0.76, 0.90)	<0.001	0.95 (0.87, 1.04)	0.271	0.85 (0.77, 0.94)	<0.001
	Model 3	0.83 (0.75, 0.90)	<0.001	0.95 (0.87, 1.04)	0.289	0.85 (0.77, 0.94)	0.001
Sleep timing (referred to sleep midpoint inside of 2:00–4:00 a.m.)	Crude	1.71 (1.25, 2.33)	0.001	1.18 (0.83, 1.67)	0.361	1.49 (1.09, 2.05)	0.013
	Model 1	1.82 (1.31, 2.51)	<0.001	1.17 (0.82, 1.66)	0.387	1.63 (1.17, 2.27)	0.004
	Model 2	1.81 (1.30, 2.50)	<0.001	1.19 (0.83, 1.71)	0.334	1.58 (1.13, 2.21)	0.008
	Model 3	1.79 (1.39, 2.49)	0.001	1.16 (0.80, 1.68)	0.423	1.57 (1.12, 2.20)	0.009
Sleep regularity (referred to irregular sleep)	Crude	1.04 (0.79, 1.39)	0.761	0.98 (0.70, 1.36)	0.884	1.03 (0.75, 1.41)	0.866
	Model 1	0.90 (0.67, 1.21)	0.474	0.92 (0.64, 1.30)	0.627	0.88 (0.63, 1.23)	0.461
	Model 2	0.91 (0.66, 1.22)	0.516	0.92 (0.64, 1.31)	0.644	0.90 (0.64, 1.25)	0.515
	Model 3	0.89 (0.66, 1.20)	0.438	0.90 (0.63, 1.31)	0.595	0.88 (0.63, 1.23)	0.444
Sleep efficiency	Crude	0.05 (0.01, 0.16)	<0.001	0.25 (0.08, 0.77)	0.015	0.17 (0.06, 0.53)	0.002
	Model 1	0.05 (0.01, 0.18)	<0.001	0.24 (0.07, 0.74)	0.014	0.20 (0.06, 0.65)	0.008
	Model 2	0.05 (0.01, 0.18)	<0.001	0.24 (0.08, 0.77)	0.017	0.20 (0.06, 0.66)	0.008
	Model 3	0.04 (0.01, 0.14)	<0.001	0.21 (0.06, 0.66)	0.008	0.18 (0.05, 0.63)	0.007

Separate multivariable binary logistic regression analyses were performed to examine the associations between sleep, sunlight exposure, and multimorbidity. Due to the small sample size of Mental-only multimorbidity (*n* = 16), we did not analyze this pattern. Adjusted odds ratios (AORs) and 95% confidence intervals were estimated using bootstrap resampling with 1,000 replications. AOR, Adjusted odds ratio; CI, Confidence interval.

With regard to sleep-related symptoms, sleep disturbance, insomnia symptoms, frequent snoring, and daytime sleepiness were significantly associated with increased odds of all multimorbidity types (*p* < 0.05). For sleep parameters, longer sleep duration demonstrated a protective effect, with each hour associated with 17% lower odds of overall multimorbidity (AOR = 0.83; 95%CI: 0.75–0.90) and 15% lower odds of mental-physical multimorbidity (AOR = 0.85, 95%CI: 0.77–0.94). Conversely, an extreme sleep midpoint was associated with 79% higher odds of overall multimorbidity (AOR = 1.79; 95%CI: 1.39–2.49) and 57% higher odds of mental-physical multimorbidity (AOR = 1.57, 95%CI: 1.12–2.20).

### 3.4 Stratified analysis by gender and age

**Figure 1:**
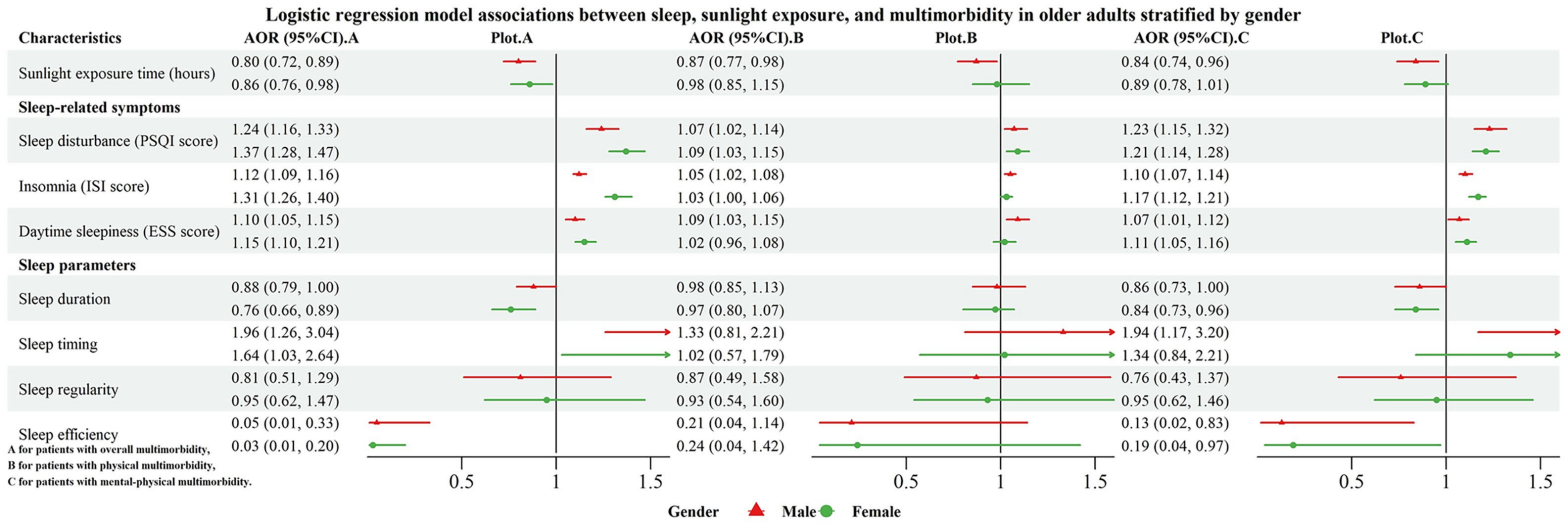
Forest plot for the associations of sleep and sunlight exposure with multimorbidity, stratified by gender.

**Figure 2:**
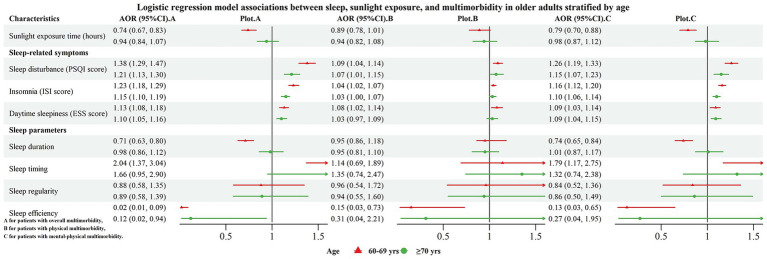
Forest plot for the associations of sleep and sunlight exposure with multimorbidity, stratified by age.

Stratified analyses by gender and age revealed notable differences in the associations (Figures 1, 2; Supplementary Tables 1, 2). Gender-stratified analyses showed that the protective association of sunlight exposure was significant only in males, with each additional hour associated with 20% lower odds of overall multimorbidity (AOR = 0.80, 95% CI: 0.72–0.89), 13% lower odds of physical-only multimorbidity (AOR = 0.87, 95% CI: 0.77–0.98), and 16% lower odds of mental-physical multimorbidity (AOR = 0.84, 95% CI: 0.74–0.96). In contrast, longer sleep duration was protective only among females, associated with 24% lower odds of overall multimorbidity (AOR = 0.76, 95% CI: 0.66–0.89) and 16% lower odds of mental-physical multimorbidity (AOR = 0.84, 95% CI: 0.73–0.96). Moreover, an extreme sleep midpoint was a risk factor observed primarily in males, associated with 1.96-fold higher odds of overall multimorbidity (AOR = 1.96, 95% CI: 1.26–3.04) and 1.64-fold higher odds of mental-physical multimorbidity (AOR = 1.80, 95% CI: 1.09–2.98).

In terms of age, stronger associations were generally observed in the younger cohort (60–69 years) compared with those aged 70 years and above. Specifically in the 60–69 years group, longer sleep duration was associated with lower odds of both overall multimorbidity (AOR = 0.71, 95% CI: 0.63–0.80) and mental-physical multimorbidity (AOR = 0.74, 95% CI: 0.65–0.84), whereas an extreme sleep midpoint was associated with increased odds of overall multimorbidity (AOR = 2.04, 95% CI: 1.37–3.04) and mental-physical multimorbidity (AOR = 1.79, 95% CI: 1.17–2.75). Similarly, the protective associations of higher sleep efficiency and longer sunlight exposure with mental-physical multimorbidity were also observed in the 60–69 years group. Although sleep-related symptoms such as sleep disturbance, clinical insomnia, and snoring were associated with increased odds in both age groups, the associations were consistently stronger among younger older adults (60–69 years).

## 4 Discussion

In the present study, we adopted a patient-centered perspective to comprehensively explore the prevalence of multimorbidity and its associations with sleep and sunlight exposure among community-dwelling older adults in Northwest China. This cross-sectional study revealed a substantial burden of multimorbidity, with nearly half of the participants affected. A key finding was that the associations of sleep-related symptoms and sunlight exposure were more pronounced in older adults with mental-physical multimorbidity, highlighting the critical interplay between mental and physical health. Although this subgroup represented a minority of the study population, it constitutes a clinically vulnerable group that appears to be particularly sensitive to sub-optimal sleep and limited sunlight exposure, with important implications for prevention and care.

Although both sleep problems and sunlight exposure were associated with multimorbidity, their association patterns differed markedly. For instance, sleep-related symptoms (e.g., sleep disturbance, insomnia, snoring, and daytime sleepiness) consistently increased the odds of all multimorbidity categories. In contrast, protective sleep parameters such as longer sleep duration and non-extreme sleep timing (sleep midpoint within 2:00–4:00 a.m.) were associated with reduced risks, particularly for mental-physical multimorbidity. Most notably, sunlight exposure was inversely associated with mental-physical multimorbidity risk. Collectively, these findings suggest that the influences of sleep and sunlight are not uniform across all multimorbidity categories, with the strongest convergence observed among individuals with co-occurring mental and physical conditions.

Previous studies have established that sunlight exposure and sleep are closely associated with mental problems and multimorbidity (14, 30). Indeed, older adults with psychiatric or multi-system patterns are at an accelerated risk of the progression to frailty and cognition impairment, as well as their co-occurrence (30). Indalino et al. (31) reported that older adults with sleep problems had a 1.34- to 1.88-fold increased risk of multimorbidity. Sindi et al. (32) utilized the sleep item from the Comprehensive Psychiatric Rating Scale, demonstrated that moderate-to-severe sleep disturbances were associated with multimorbidity. Our findings are consistent with these studies and extend the existing evidence by demonstrating that specific sleep-related symptoms, including poor sleep quality, insomnia, snoring, and daytime sleepiness, were significantly associated with multimorbidity. With respect to sunlight exposure, we observed the most pronounced protective effect against mental-physical multimorbidity among males and younger older adults aged 60–69 years. These findings further substantiate the potential benefits of sunlight exposure for individuals with co-occurring mental and physical conditions, aligning with prior literature indicating that sunlight exposure in natural environments confers additional mental health benefits, such as reductions in depression and anxiety symptoms associated with walking in sunshine (33). Importantly, other studies, such as Ma et al. (17), reported a J-shaped association between outdoor light exposure and dementia risk, wherein risk increased markedly at low exposure levels but rose more gradually at higher exposure levels. This pattern suggests that the relationship between sunlight exposure time and health outcomes, including multimorbidity may be non-linear.

The relationship between sleep and multimorbidity is probably bidirectional. For instance, short sleep duration (< 6 h) led to a 1.49-fold increased risk of incident multimorbidity over a 3-year follow-up, whereas multimorbidity was associated with a 1.39-fold higher risk of short sleep duration after full adjustment (34). Another prospective cohort study from the UK Biobank, which used healthy sleep pattern score encompassing five dimensions (i.e., early chronotype, sleep 7–8 h/d, free of insomnia, no snoring, and no frequent excessive daytime sleepiness), found that each one-point increase was associated with an adjusted hazard ratio (HR) of 0.93 (95% CI: 0.91–0.95) for cardiometabolic multimorbidity (CMM) in older adults (35). Zhou et al. (36) used data from the China Health and Retirement Longitudinal Study also demonstrated similar conclusions. They found that both short sleep duration ≤ 5 h and 5–7 restless days per week were significantly associated with a higher risk of multimorbidity progression. Other studies have demonstrated a mediating role of mental health status in the association between sleep duration and cognitive function among older adults with multimorbidity, further indicating that interventions targeting sleep and mental health may be effective in this population (37). Therefore, governments and health service systems should prioritize improving the management of people with multimorbidity by using a more patient-centered approach that promote the integrated treatment of physical and mental health conditions (38).

Furthermore, our stratified analyses indicated that the associations were moderated by gender and age. Males appeared to derive greater benefit from prolonged sunlight exposure but were also more vulnerable to the adverse effects associated with an extreme sleep midpoint. Conversely, the protective effect of extended sleep duration was more pronounced in females. Additionally, compared with older adults aged 70 years and older, younger older adults (60–69 years) were more susceptible to the influence of both limited sunlight exposure and poor sleep than their older counterparts. These findings emphasize that prevention and intervention strategies should be tailored according to gender and age. For instance, promoting adequate sunlight exposure may be particularly relevant for males and younger older adults, whereas interventions aimed at extending sleep duration may be especially beneficial for females.

Despite the evidence provided by this study, several limitations should be acknowledged. First, sunlight exposure was assessed using self-reported time spent outdoors, which does not capture objective measures of light intensity or duration at the ocular level. Second, the cross-sectional nature of our study precludes capturing any causal inference, as it collects exposure and outcome data simultaneously, thereby preventing determination of whether poor sleep or limited sunlight exposure precipitated the development of multimorbidity, or whether, conversely, multimorbidity leads to poorer sleep and reduced sunlight exposure. Third, multimorbidity subtypes were analyzed as separate multivariable binary logistic regression models, which may limit direct comparability with studies adopting typology-based or multinomial classification approaches. In addition, the number of participants with mental-only multimorbidity was very small (n = 16), precluding reliable statistical analysis of this subgroup. Finally, the sample was primarily drawn from specific regions with relatively low socioeconomic status, which may affect the generalizability of our results to other populations.

## 5 Conclusion

Herein, we reported cross-sectional associations between sunlight exposure, sleep, and multimorbidity among older adults in Northwest China, with the most robust associations observed for the mental-physical multimorbidity category, which refers to the concurrent presence of physical conditions and mental illness. These findings underscore a critical need to integrate routine mental health screening into the management of multimorbidity for healthcare practitioners in primary care settings. Importantly, although mental-physical multimorbidity affected a minority of older adults in this population, this subgroup represents a particularly vulnerable group that appears to be more sensitive to modifiable lifestyle-related factors. For older adults who identified with co-occurring mental and physical conditions, targeting interventions focusing on evidence-based sleep management strategies like improving sleep hygiene and promoting appropriate sunlight exposure, may represent feasible and patient-centered approaches to preventing the progression or alleviating the burden of mental-physical multimorbidity.

## Data Availability

The original contributions presented in the study are included in the article/Supplementary material, further inquiries can be directed to the corresponding authors.

## References

[1] Skou ST Mair FS Fortin M Guthrie B Nunes BP Miranda JJ . Multimorbidity. Nat Rev Dis Primers. (2022) 8:48. doi: 10.1038/s41572-022-00376-4, 35835758 PMC7613517

[2] Salive ME. Multimorbidity in older adults. Epidemiol Rev. (2013) 35:75–83. doi: 10.1093/epirev/mxs009, 23372025

[3] Wei MY Mukamal KJ. Multimorbidity and mental health-related quality of life and risk of completed suicide. J Am Geriatr Soc. (2019) 67:511–9. doi: 10.1111/jgs.15678, 30471103 PMC6402970

[4] Prados-Torres A Calderón-Larrañaga A Hancco-Saavedra J Poblador-Plou B van den Akker M. Multimorbidity patterns: a systematic review. J Clin Epidemiol. (2014) 67:254–66. doi: 10.1016/j.jclinepi.2013.09.021, 24472295

[5] Zhao Y Zhang P Oldenburg B Hall T Lu S Haregu TN . The impact of mental and physical multimorbidity on healthcare utilization and health spending in China: a nationwide longitudinal population-based study. Int J Geriatr Psychiatry. (2021) 36:500–10. doi: 10.1002/gps.5445, 33037674

[6] Pan T Mercer SW Zhao Y McPake B Desloge A Atun R . The association between mental-physical multimorbidity and disability, work productivity, and social participation in China: a panel data analysis. BMC Public Health. (2021) 21:376. doi: 10.1186/s12889-021-10414-7, 33602174 PMC7890601

[7] Taloyan M Alinaghizadeh H Wettermark B Jan Hasselström JH Bertilson BC. Physical-mental multimorbidity in a large primary health care population in Stockholm County, Sweden. Asian J Psychiatr. (2023) 79:103354. doi: 10.1016/j.ajp.2022.103354, 36463815

[8] Halstead S Sartorius N Every-Palmer S Siddiqi N de Girolamo G Siskind D . Physical multimorbidity and mental illness: a global challenge. Aust N Z J Psychiatry. (2024) 58:293–6. doi: 10.1177/00048674241235587, 38517131

[9] Ishida M Hulse ES Mahar RK Gunn J Atun R McPake B . The joint effect of physical multimorbidity and mental health conditions among adults in Australia. Prev Chronic Dis. (2020) 17:E157. doi: 10.5888/pcd17.200155, 33301391 PMC7769083

[10] Ahmed W Muhammad T Muneera K. Prevalence of early and late onset of chronic diseases and multimorbidity and its association with physical, mental and functional health among older Indian adults. BMC Geriatr. (2023) 23:563. doi: 10.1186/s12877-023-04264-8, 37710170 PMC10502995

[11] Pizzol D Trott M Butler L Barnett Y Ford T Neufeld SA . Relationship between severe mental illness and physical multimorbidity: a meta-analysis and call for action. BMJ Ment Health. (2023) 26:1–5. doi: 10.1136/bmjment-2023-300870, 37907331 PMC10619039

[12] Gaulin M Simard M Candas B Lesage A Sirois C. Combined impacts of multimorbidity and mental disorders on frequent emergency department visits: a retrospective cohort study in Quebec, Canada. CMAJ. (2019) 191:E724–32. doi: 10.1503/cmaj.181712, 31266786 PMC6606417

[13] Zhang J Chen L Zhang S Cai M Zou H Vaughn MG . Associations of sleep patterns with dynamic trajectory of cardiovascular multimorbidity and mortality: a multistate analysis of a large cohort. J Am Heart Assoc. (2023) 12:e029463. doi: 10.1161/jaha.123.029463, 37776189 PMC10727256

[14] Song Y Chen L Liu Y. Association between nap time, nighttime sleep, and multimorbidity in Chinese older adults: a cross-sectional study. BMC Geriatr. (2025) 25:151. doi: 10.1186/s12877-025-05807-x, 40045201 PMC11881392

[15] Smith L Shin JI Jacob L Schuch F Oh H Tully MA . Association between physical multimorbidity and sleep problems in 46 low- and middle-income countries. Maturitas. (2022) 160:23–31. doi: 10.1016/j.maturitas.2022.01.007, 35550705

[16] Zhou Y Jin Y Zhu Y Fang W Dai X Lim C . Sleep problems associate with multimorbidity: a systematic review and meta-analysis. Public Health Rev. (2023) 44:1605469. doi: 10.3389/phrs.2023.1605469, 37383367 PMC10293634

[17] Ma LZ Ma YH Ou YN Chen SD Yang L Dong Q . Time spent in outdoor light is associated with the risk of dementia: a prospective cohort study of 362094 participants. BMC Med. (2022) 20:132. doi: 10.1186/s12916-022-02331-2, 35462547 PMC9036798

[18] Burns AC Windred DP Rutter MK Olivier P Vetter C Saxena R . Day and night light exposure are associated with psychiatric disorders: an objective light study in >85,000 people. Nature Mental Health. (2023) 1:853–62. doi: 10.1038/s44220-023-00135-8

[19] Burns AC Saxena R Vetter C Phillips AJK Lane JM Cain SW. Time spent in outdoor light is associated with mood, sleep, and circadian rhythm-related outcomes: a cross-sectional and longitudinal study in over 400,000 UK biobank participants. J Affect Disord. (2021) 295:347–52. doi: 10.1016/j.jad.2021.08.056, 34488088 PMC8892387

[20] Walker WH 2nd Walton JC DeVries AC Nelson RJ. Circadian rhythm disruption and mental health. Transl Psychiatry. (2020) 10:28. doi: 10.1038/s41398-020-0694-0, 32066704 PMC7026420

[21] Koronowski KB Sassone-Corsi P. Communicating clocks shape circadian homeostasis. Science. (2021) 371:371. doi: 10.1126/science.abd0951, 33574181 PMC8123919

[22] Whitmore D Foulkes NS Sassone-Corsi P. Light acts directly on organs and cells in culture to set the vertebrate circadian clock. Nature. (2000) 404:87–91. 10716448 10.1038/35003589

[23] Buysse DJ. Sleep health: can we define it? Does it matter? Sleep. (2014) 37:9–17. doi: 10.5665/sleep.3298, 24470692 PMC3902880

[24] Wang S Feng M Fang Y Lv L Sun G Cheng S . Effects of chronotype on antidepressant treatment and symptoms in patients with depression: a multicenter, parallel, controlled study. BMC Psychiatry. (2023) 23:277. doi: 10.1186/s12888-023-04721-z, 37081401 PMC10120275

[25] Vitiello MV Zhu W Von Korff M Wellman R Morin CM Yeung K . Long-term improvements in sleep, pain, depression, and fatigue in older adults with comorbid osteoarthritis pain and insomnia. Sleep. (2022) 45:zsab231. doi: 10.1093/sleep/zsab231, 34516646 PMC8842298

[26] Wang YT Hsu NW Lin CH Chen HC. Concurrence of insomnia and daytime sleepiness predicted 9-year mortality risk in community-dwelling older adults: the Yilan study, Taiwan. J Gerontol A Biol Sci Med Sci. (2023) 78:2371–81. doi: 10.1093/gerona/glad201, 37596845

[27] Shin C Park MH Lee SH Ko YH Kim YK Han KM . Usefulness of the 15-item geriatric depression scale (GDS-15) for classifying minor and major depressive disorders among community-dwelling elders. J Affect Disord. (2019) 259:370–5. doi: 10.1016/j.jad.2019.08.053, 31470180

[28] Kroenke K Spitzer RL Williams JB Monahan PO Löwe B. Anxiety disorders in primary care: prevalence, impairment, comorbidity, and detection. Ann Intern Med. (2007) 146:317–25. doi: 10.7326/0003-4819-146-5-200703060-00004, 17339617

[29] Wang X Zheng N Yin M. Multimorbidity patterns and depression: bridging epidemiological associations with predictive analytics for risk stratification. Healthcare (Basel). (2025) 13:1458. doi: 10.3390/healthcare13121458, 40565485 PMC12193055

[30] Wang S Li Q Hu J Chen Q Wang S Xue QL . Association of multimorbidity patterns and order of physical frailty and cognitive impairment occurrence: a prospective cohort study. Age Ageing. (2025) 54:afaf101. doi: 10.1093/ageing/afaf101, 40263943 PMC12014529

[31] Idalino SCC Canever JB Cândido LM Wagner KJP de Souza Moreira B Danielewicz AL . Association between sleep problems and multimorbidity patterns in older adults. BMC Public Health. (2023) 23:978. doi: 10.1186/s12889-023-15965-5, 37237275 PMC10224570

[32] Sindi S Pérez LM Vetrano DL Triolo F Kåreholt I Sjöberg L . Sleep disturbances and the speed of multimorbidity development in old age: results from a longitudinal population-based study. BMC Med. (2020) 18:382. doi: 10.1186/s12916-020-01846-w, 33280611 PMC7720467

[33] Kelly P Williamson C Niven AG Hunter R Mutrie N Richards J. Walking on sunshine: scoping review of the evidence for walking and mental health. Br J Sports Med. (2018) 52:800–6. doi: 10.1136/bjsports-2017-098827, 29858467

[34] Wang X Wang R Zhang D. Bidirectional associations between sleep quality/duration and multimorbidity in middle-aged and older people Chinese adults: a longitudinal study. BMC Public Health. (2024) 24:708. doi: 10.1186/s12889-024-17954-8, 38443848 PMC10916205

[35] He L Ma T Li J Luo Y Zhang G Cheng X . Adherence to a healthy sleep pattern and incidence of cardiometabolic multimorbidity among hypertensive patients: a prospective study of UK biobank. Sleep. (2022) 45:zsac141. doi: 10.1093/sleep/zsac141, 35738866 PMC9548671

[36] Zhou Y Ni Y Jones M Dai X Lim CCW Zhu A . Sleep behaviors and progression of multimorbidity in middle-aged and older adults: a prospective cohort study from China. J Gerontol A Biol Sci Med Sci. (2023) 78:1871–80. doi: 10.1093/gerona/glad087, 36943283

[37] Jia M Liu X Da X. Association between nighttime sleep duration and cognitive function in middle-aged and older adult patients with multimorbidity: the mediating role of depression. Front Public Health. (2025) 13:1576629. doi: 10.3389/fpubh.2025.1576629, 40703181 PMC12283587

[38] Seakamela KP Mashaba RG Ntimana CB Kabudula CW Sodi T. Multimorbidity management: a scoping review of interventions and health outcomes. Int J Environ Res Public Health. (2025) 22:770. doi: 10.3390/ijerph22050770, 40427886 PMC12111452

