# Supplementary material for: Association between sleep, sunlight exposure, and multimorbidity in older adults with and without mental illness
Source: Front Public Health. 2026 Mar 10;14:1751563. doi: 10.3389/fpubh.2026.1751563 (PMC13008644; doi:10.3389/fpubh.2026.1751563)
Supplement: Supplementary file 1 [file Table_1.docx]

**SUPPLEMENTARY TABLE 1** The gender-stratified associations between sleep, sunlight exposure, and multimorbidity in older adults.

| Variables | Overall multimorbidity  (≥2 chronic conditions) | | Physical-only multimorbidity  (≥2 physical conditions) | | Mental-physical multimorbidity  (co-occurrence of physical and mental conditions) | |
| --- | --- | --- | --- | --- | --- | --- |
|  | Male | Female | Male | Female | Male | Female |
| Sunlight exposure time (hours) | 0.80 (0.72-0.89) | 0.86 (0.76-0.98) | 0.87 (0.77-0.98) | 0.98 (0.85-1.15) | 0.84 (0.74-0.96) | 0.89 (0.78-1.01) |
| Sleep disturbance (PSQI score) | 1.24 (1.16-1.33) | 1.37 (1.28-1.47) | 1.07 (1.02-1.14) | 1.09 (1.03-1.15) | 1.23 (1.15-1.32) | 1.21 (1.14-1.28) |
| Insomnia (ISI score) | 1.12 (1.09-1.16) | 1.31 (1.26-1.40) | 1.05 (1.02-1.08) | 1.03 (1.00-1.06) | 1.10 (1.07-1.14) | 1.17 (1.12-1.21) |
| Sometimes snoring (referred to never or rare snoring) | 3.34 (2.07-5.37) | 3.71 (2.27-6.06) | 2.38 (1.42-4.00) | 1.99 (1.16-3.42) | 2.53 (1.39-4.62) | 2.25 (1.43-3.53) |
| Often snoring (referred to never or rare snoring) | 5.91 (3.16-11.03) | 6.25 (2.67-14.64) | 2.76 (1.52-5.01) | 2.88 (1.47-5.63) | 3.84 (2.02-7.31) | 2.24 (1.24-4.04) |
| Daytime sleepiness (ESS score) | 1.10 (1.05-1.15) | 1.15 (1.10-1.21) | 1.09 (1.03-1.15) | 1.02 (0.96-1.08) | 1.07 (1.01-1.12) | 1.11 (1.05-1.16) |
| Sleep duration | 0.88 (0.79-1.00) | 0.76 (0.66-0.89) | 0.98 (0.85-1.13) | 0.97 (0.80-1.07) | 0.86 (0.73-1.00) | 0.84 (0.73-0.96) |
| Sleep timing (referred to sleep midpoint inside of 2:00-4:00 AM) | 1.96 (1.26-3.04) | 1.64 (1.03-2.64) | 1.33 (0.81-2.21) | 1.02 (0.57-1.79) | 1.94 (1.17-3.20) | 1.34 (0.84-2.21) |
| Sleep regularity (referred to irregular sleep) | 0.81 (0.51-1.29) | 0.95 (0.62-1.47) | 0.87 (0.49-1.58) | 0.93 (0.54-1.60) | 0.76 (0.43-1.37) | 0.95 (0.62-1.46) |
| Sleep efficiency | 0.05 (0.01-0.33) | 0.03 (0.01-0.20) | 0.21 (0.04-1.14) | 0.24 (0.04-1.42) | 0.13 (0.02-0.83) | 0.19 (0.04-0.97) |

Note: Effect size is presented as adjusted odds ratio (AOR) with its 95% confidence interval (CI). The analysis was performed using the full-adjusted model.

**SUPPLEMENTARY TABLE 2** The age-stratified associations between sleep, sunlight exposure, and multimorbidity in older adults.

| Variables | Overall multimorbidity (≥2 chronic conditions) | | Physical-only multimorbidity  (≥2 physical conditions) | | Mental-physical multimorbidity  (co-occurrence of physical and mental conditions) | |
| --- | --- | --- | --- | --- | --- | --- |
|  | 60-69y | ≥ 70y | 60-69y | ≥ 70y | 60-69y | ≥ 70y |
| Sunlight exposure time (hours) | 0.74 (0.67-0.83) | 0.94 (0.84-1.07) | 0.89 (0.78-1.01) | 0.94 (0.82-1.08) | 0.79 (0.70-0.88) | 0.98 (0.87-1.12) |
| Sleep disturbance (PSQI score) | 1.38 (1.29-1.47) | 1.21 (1.13-1.30) | 1.09 (1.04-1.14) | 1.07 (1.01-1.15) | 1.26 (1.19-1.33) | 1.15 (1.07-1.23) |
| Insomnia (ISI score) | 1.23 (1.18-1.29) | 1.15 (1.10-1.19) | 1.04 (1.02-1.07) | 1.03 (1.00-1.07) | 1.16 (1.12-1.20) | 1.10 (1.06-1.14) |
| Sometimes snoring (referred to never or rare snoring) | 3.54 (2.27-5.52) | 3.47 (1.94-6.20) | 2.07 (1.22-3.52) | 2.21 (1.22-4.01) | 2.56 (1.63-4.02) | 2.13 (1.22-3.72) |
| Often snoring (referred to never or rare snoring) | 5.48 (3.02-9.95) | 6.35 (2.67-15.10) | 2.95 (1.60-5.47) | 2.12 (1.08-4.16) | 2.70 (1.50-4.84) | 3.29 (1.58-6.87) |
| Daytime sleepiness (ESS score) | 1.13 (1.08-1.18) | 1.10 (1.05-1.16) | 1.08 (1.02-1.14) | 1.03 (0.97-1.09) | 1.09 (1.03-1.14) | 1.09 (1.04-1.15) |
| Sleep duration | 0.71 (0.63-0.80) | 0.98 (0.86-1.12) | 0.95 (0.86-1.18) | 0.95 (0.81-1.10) | 0.74 (0.65-0.84) | 1.01 (0.87-1.17) |
| Sleep timing (referred to sleep midpoint inside of 2:00-4:00 AM) | 2.04 (1.37-3.04) | 1.66 (0.95-2.90) | 1.14 (0.69-1.89) | 1.35 (0.74-2.47) | 1.79 (1.17-2.75) | 1.32 (0.74-2.38) |
| Sleep regularity (referred to irregular sleep) | 0.88 (0.58-1.35) | 0.89 (0.58-1.39) | 0.96 (0.54-1.72) | 0.94 (0.55-1.60) | 0.84 (0.52-1.36) | 0.86 (0.50-1.49) |
| Sleep efficiency | 0.02 (0.01-0.09) | 0.12 (0.02-0.94) | 0.15 (0.03-0.73) | 0.31 (0.04-2.21) | 0.13 (0.03-0.65) | 0.27 (0.04-1.95) |

Note: Effect size is presented as adjusted odds ratio (AOR) with its 95% confidence interval (CI). The analysis was performed using the full-adjusted model.
